# Psychosocial Outcomes of IPV Interventions in Women With and Without Probable IPV-Related Brain Injury

**DOI:** 10.3390/brainsci16080836

**Published:** 2026-08-06

**Authors:** Brigitta M. Beck, Michelle M. Pebole, Kristiana D. Carrasquillo, Colin T. Mahoney, Katherine M. Iverson

**Affiliations:** 1 Department of Psychology, University of Colorado, Colorado Springs, CO 80918, USA; bbeck2@uccs.edu; 2 Translational Research Center for TBI and Stress Disorders National Network Center, VA Boston Healthcare System, Boston, MA 02215, USA; michelle.pebole@va.gov; 3 Women’s Health Sciences Division of the National Center for PTSD, VA Boston Healthcare System, Boston, MA 02215, USA; 4 Department of Psychology, The University of New Mexico, Albuquerque, NM 87106, USA; kristic@unm.edu (K.D.C.);; 5 Center on Alcohol, Substance Use, and Addictions (CASAA), The University of New Mexico, Albuquerque, NM 87106, USA; 6 Department of Psychiatry, Chobanian and Avedisian School of Medicine, Boston University, Boston, MA 02118, USA

**Keywords:** intimate partner violence, concussion, traumatic brain injury, psychosocial outcomes, women veterans, healthcare interventions

## Abstract

**Highlights:**

**What are the main findings?**

- Women with and without a history of probable IPV-related traumatic brain injury (TBI) showed similar improvements in self-efficacy, empowerment, patient activation, depression, and resilience following brief healthcare-based IPV interventions.
- Probable IPV-related TBI was associated with differences in valued living and anxiety outcomes, suggesting that TBI history may affect specific aspects of recovery.

**What are the implications of the main findings?**

- Brief trauma-informed IPV interventions may benefit women regardless of IPV-related TBI history, supporting their use in healthcare settings.
- Brain injury-informed strategies and cognitive accommodations may help improve valued living and anxiety among women with IPV-related TBI histories.

**Abstract:**

**Background:** Between 33% and 75% of women who experience intimate partner violence (IPV) sustain an IPV-related traumatic brain injury (TBI). Although IPV-related TBI is associated with adverse psychosocial outcomes, its impact on response to healthcare-based IPV interventions remains unclear. **Methods:** Using data from a randomized clinical trial within the Veterans Health Administration, we examined psychosocial outcomes among 60 woman veterans with past-year IPV experiences participating in two brief IPV interventions: Enhanced Care As Usual (ECAU) and Recovering from IPV through Strengths and Empowerment (RISE). **Results:** At pretreatment, 21 women (35%) reported a history of probable IPV-related TBI. Multilevel modeling revealed no significant main or interaction effects of TBI history for self-efficacy, empowerment, patient activation, depression, physical health symptoms, or resilience. However, there was a significant interaction of time and probable IPV-related TBI history for valued living, such that women with probable IPV-related TBI history demonstrated greater initial gains followed by slight posttreatment decline; *F*(2, 76.55) = 2.96, *p* = 0.049. There was also a significant interaction between probable IPV-related TBI history, treatment condition, and pretreatment anxiety symptoms for anxiety symptoms as an outcome, such that women with probable IPV-related TBI history in the RISE condition, who had more severe anxiety symptoms at pretreatment, reported higher anxiety symptoms on average across the study period than women without probable IPV-related TBI history in RISE; *F*(1, 48) = 5.04, *p* = 0.029. Furthermore, there was a significant interaction of probable IPV-related TBI history and pretreatment physical health symptoms for physical health symptoms as an outcome, such that women with probable IPV-related TBI histories maintained higher physical symptoms from pretreatment to the last follow-up assessment; *F*(1, 134) = 8.99, *p* = 0.004. **Conclusions:** No evidence of differential treatment response was detected for women with and without probable IPV-related TBI history. Findings related to valued living and anxiety suggest areas where additional support may improve implementation of brief healthcare-based IPV interventions.

## 1. Introduction

Intimate Partner Violence (IPV) is a significant public health issue, commonly defined as physical, sexual, and/or psychological violence and aggression by a current or former intimate partner [1]. According to the 2023/2024 National Intimate Partner and Sexual Violence Survey, approximately 1 in 3 women in the United States experience IPV during their lifetime [2]. Among women experiencing IPV, traumatic brain injury (TBI) is a common but often underrecognized consequence of violence. IPV-related brain injury may result from blunt-force trauma to the head, neck, or face; repetitive head impacts and their cumulative effects; and hypoxic or anoxic mechanisms associated with non-fatal strangulation, which frequently occur repeatedly and may intensify over time [3,4,5,6,7]. Given the significant psychosocial and health consequences associated with IPV-related TBI, this injury may further shape recovery trajectories and responses to healthcare-based support among women who experience IPV.

Women who experience IPV often face a broad range of mental, physical, and psychosocial consequences. IPV is associated with elevated symptoms of anxiety, depression, post-traumatic stress disorder (PTSD), and substance use disorders, as well as suicidal ideation [8,9,10,11,12]. Beyond these mental health symptoms, IPV can also disrupt women’s broader recovery-related outcomes, including sense of empowerment, self-efficacy, and engagement in meaningful life activities (i.e., valued living). These constructs are important because recovery from IPV involves not only attenuation of symptom distress, but also rebuilding autonomy, confidence, and participation in valued areas of life [13,14].

Prior research on women experiencing IPV suggests that greater self-care, agency, and self-efficacy may function as resilience-related processes that support adjustment following IPV [15]. More recent longitudinal work further supports the importance of self-efficacy, showing that psychological distress stemming from IPV predicted lower self-efficacy over time, which was then associated with greater subsequent IPV experiences among women [16]. Relatedly, IPV experiences have been associated with lower self-efficacy, which in turn predicted reduced engagement in personally meaningful life domains years later [17]. Valued living, or engagement in personally meaningful life domains, may therefore represent an important recovery-oriented outcome following IPV. Additionally, patient activation, defined as the knowledge, confidence, and skills needed to manage one’s health and healthcare, may also be particularly relevant for women experiencing IPV, given the complex physical, psychological, and safety-related needs that may shape engagement with healthcare-based interventions [18]. Together, these findings suggest that IPV is associated with disruptions in both mental health and broader aspects of psychosocial functioning and represent important treatment targets. This highlights the importance of examining the impacts of IPV interventions on important psychosocial outcomes such as self-efficacy, empowerment, patient activation, valued living, depression, physical health symptoms, and resilience among women who experience IPV. Importantly, IPV frequently co-occurs with other forms of injury and health risk. One particularly relevant but often underrecognized consequence is TBI, which may further compound women’s mental disorder symptoms, functional impairment, and ability to engage in recovery-oriented behaviors [19,20].

In the context of IPV, TBIs frequently result from blows to the head, face, and neck; violent shaking; repetitive head impacts; and hypoxic or anoxic injury associated with non-fatal strangulation, all of which can produce both acute and cumulative neurologic insult [3,7,21,22]. Approximately 33% to 75% of women who experience IPV have endured at least one TBI from an intimate partner, underscoring the scope of this issue [20,23]. The consequences of TBI in this population span cognitive, emotional, and behavioral domains, with common impairments including executive dysfunction, difficulties with attention and memory, and challenges with emotion regulation [3,20,24,25]. These deficits may substantially interfere with daily functioning, including the ability to maintain relationships, maintain stable employment, and navigate complex systems of care, potentially impacting patient activation and hindering one’s ability to effectively engage in healthcare decision-making [3,26]. Importantly, such impairments are closely tied to broader psychosocial functioning, potentially exacerbating vulnerability and limiting recovery. Given these factors, TBI may play a key role in shaping how individuals respond to psychosocial interventions, highlighting the importance of considering TBI history when evaluating treatment outcomes among women who experience IPV. Although findings from the broader TBI literature suggest that individuals with a history of TBI may experience difficulties with treatment response and skill acquisition [27,28], emerging evidence suggests that individuals with IPV-related TBI can experience meaningful clinical improvement following healthcare-based IPV interventions. Recovering from IPV through Strengths and Empowerment (RISE) is a brief, clinician-delivered, survivor-centered counseling intervention based in empowerment principles, trauma-informed care, and motivational interviewing [29]. Designed as an individualized and modular treatment, RISE allows participants to focus on concerns most relevant to their recovery from IPV while emphasizing choice and voice throughout treatment [29]. In the parent randomized clinical trial (RCT), RISE was compared with an Enhanced Care As Usual (ECAU) intervention, a single-session advocacy-based intervention that incorporated psychoeducation, safety planning, validation, resources and referrals to services [29]. Findings from the trial demonstrated that participants receiving RISE experienced greater gains in empowerment and self-efficacy relative to ECAU, while participants in both intervention arms showed similar improvements over time in depression, valued living, and reductions in IPV [29].

Although these findings support the psychosocial benefits of RISE and advocacy-based ECAU, it remains unclear how a history of probable IPV-related TBI may impact outcomes within such healthcare-based IPV interventions. Previous research has demonstrated that RISE and ECAU are similarly effective in improving safety outcomes among women with probable IPV-related TBI such that women with a probable IPV-related TBI history experienced larger reductions in the frequency of physical IPV and similar reductions in sexual IPV across both treatment conditions compared to women without a probable IPV-related TBI history [30]. These findings suggest that brief healthcare-based interventions may result in improvements in safety outcomes for women with and without a history of probable IPV-related TBI. Despite these encouraging findings, the associations between a probable IPV-related TBI history and psychosocial treatment response have not yet been explored. Examining whether probable IPV-related TBI moderates intervention outcomes could clarify which women benefit most from brief healthcare-based interventions, such as RISE and advocacy-based care as usual, and could inform treatment matching and tailoring.

The current study addresses this gap in examining the impact of probable IPV-related TBI history on psychosocial outcomes through a secondary analysis of data from a larger randomized clinical trial comparing two brief healthcare-based IPV interventions. We aim to examine if there are differences in treatment response from women with histories of probable IPV-related TBI in comparison to those without these histories. Based on the prior literature demonstrating comparable improvements in safety-related outcomes among women with and without probable IPV-related TBI histories following RISE and ECAU interventions [30], we hypothesized that there would be minimal differences in treatment response across broader psychosocial domains.

## 2. Methods and Materials

### 2.1. Study Design

The present study was a secondary analysis of data from a parent RCT comparing RISE with advocacy-based ECAU [29] for detailed description of the parent trial. In brief, participants were recruited between October 2018 and September 2020 from Veterans Health Administration (VHA) clinics in the northeastern United States. Eligibility criteria included: (1) identifying as a woman, (2) reporting past-year IPV, including at least one incident of physical, psychological, and/or sexual IPV, (3) receipt of VHA healthcare services within the past year, (4) age 18 years or older, and (5) willingness to have sessions audio-recorded. Exclusion criteria included current bipolar or psychotic disorder symptoms/diagnosis and suicidal or homicidal ideation with intent. Psychosocial outcomes were assessed via self-report questionnaires at pretreatment, 10 weeks, and 14 weeks. The study was approved by the VA Boston Healthcare System Institutional Review Board, and all participants provided written informed consent (ClinicalTrials.gov identifier: NCT03261700). Participant safety monitoring, adverse event procedures, crisis response protocols, and referral pathways followed procedures approved by the VA Boston Healthcare System Institutional Review Board and a Data Safety Monitoring Board that was established for the parent trial [29] and the adverse events section of the corresponding ClinicalTrials.gov registration.

### 2.2. Participants and Procedures

Sixty participants were randomized to either RISE or ECAU; 59 were included in our modified intent-to-treat analyses because one participant had no follow-up data. This participant, who was randomized to RISE, was the only case with missing outcome data.

As previously reported, participants were on average 39.1 years old (SD = 11.9), 45.0% identified as a racial minority, and 56.7% reported an annual household income above USD 45,000. Approximately 45.0% of the sample reported ongoing involvement with a violent partner at pretreatment.

### 2.3. Measures

#### 2.3.1. Participant Characteristics

Sociodemographic characteristics were collected via self-report questionnaires.

#### 2.3.2. History of Probable IPV-Related TBI

Probable IPV-related TBI history was assessed using a modified version of the validated VA TBI screening tool [31] (available from the authors upon request). This tool assessed lifetime IPV-related TBI since the age of 18 and did not have to be associated with the abusive relationship that led them to join the trial. The modification was for item 1, in which examples of deployment-related events that may increase risk for TBI (e.g., blasts, combat) were replaced with IPV-related head events. Women were considered to have experienced a probable IPV-related TBI if they reported experiencing at least 1 of 8 head events by violence from an intimate partner (being hit in the head; being pushed/shoved into objects; sustaining dental or jaw injuries; eye or ear injuries; strangulation/choking; being thrown down the stairs; or other trauma to the head, neck, or face). Consistent with the VA/Department of Defense clinical guidelines [32], women were considered to screen positive for probable IPV-related TBI history if they reported that the head events were associated with loss of consciousness, altered consciousness (i.e., being dazed or confused), post-traumatic amnesia (i.e., not remembering events before or after the injury), concussion, or head injury. This measure has been used previously with samples of women veterans [33,34], and is based on a VA validated and recommended screening tool, though it has not been formally validated itself.

### 2.4. Psychosocial Health Outcomes

Psychosocial health outcomes were evaluated using validated self-report measures administered at pretreatment, 10 weeks, and 14 weeks.

Self-efficacy was measured using the General Self-Efficacy Scale (GSES), a 10-item instrument assessing perceived confidence in managing difficult situations and coping with challenges [35]. Items (e.g., “I can always manage to solve difficult problems if I try hard enough”) are rated on a 4-point Likert scale ranging from 1 (“not at all true”) to 4 (“exactly true”). Scores are summed, with higher scores indicating greater self-efficacy. The scale has demonstrated strong reliability and construct validity across diverse populations [35]. Internal consistency in the current sample at each time point was as follows: α = 0.88 (pretreatment), 10-week follow-up (α = 0.89), and 14-week follow-up (α = 0.88).

Empowerment was assessed with the Personal Progress Scale–Revised (PPS-R), which measures women’s perceptions of personal strength, agency, and control over their lives [36]. Items are rated on a 7-point scale (1 = almost never; 7 = almost always) assessing agreement with statements related to self-worth, decision-making, and personal power. Items are summed for a total score. Higher scores reflect greater empowerment. Prior studies support the scale’s reliability and validity [36]. Internal consistency in the current sample at pretreatment was α = 0.89 (pretreatment), 10-week follow-up (α = 0.90), and 14-week follow-up (α = 0.91).

Patient activation was measured using the Patient Activation Measure-13 (PAM-13), which evaluates individuals’ knowledge, confidence, and skills for managing their health and healthcare [37]. Items (e.g., “I am confident that I can take actions that will help prevent or minimize some symptoms or problems associated with my health condition”) are rated on a 4-point scale from 1 (“strongly disagree”) to 4 (“strongly agree”), with an additional “not applicable” option. Total scores are calculated by dividing the raw score by the number of items answered and multiplying by 13. Scores are transformed to a 0–100 scale. Higher scores indicate greater activation in health self-management. The PAM-13 demonstrated good reliability and construct validity [37]. Internal consistency in the current sample at each time point was as follows: α = 0.88 (pretreatment), 10-week follow-up (α = 0.91), and 14-week follow-up (α = 0.90).

Valued living was assessed using the Valued Living Questionnaire (VLQ), which examines the extent to which individuals live consistently with personally important values across life domains [38]. Higher scores indicate greater alignment between personal values and daily behavior. The questionnaire assesses both the importance of values domains and consistency of actions with those values using 10-point Likert-type scales. Participants use a 1–10 scale to rate their values and how consistently they have lived in accordance with each value over the past week. Responses on both scales are multiplied for a valued living composite score. The VLQ has shown adequate construct validity and internal consistency reliability [38]. Internal consistency in the current sample at each time point was as follows: α = 0.84 (pretreatment), 10-week follow-up (α = 0.83), and 14-week follow-up (α = 0.85).

Depressive symptoms were assessed using the Center for Epidemiologic Studies Depression Scale (CES-D), a 20-item measure evaluating the frequency of depressive symptoms experienced during the past week [39]. Items (e.g., “I felt depressed”) are rated on a 4-point scale ranging from 0 (“rarely or none of the time”) to 3 (“most or all of the time”). Higher scores indicate greater depressive symptom severity. The CES-D has demonstrated strong reliability and validity in community and clinical populations [39]. Internal consistency in the current sample at each time point was as follows: α = 0.90 (pretreatment), 10-week follow-up (α = 0.90), and 14-week follow-up (α = 0.93).

Anxiety symptoms were measured using the Anxiety subscale of the Depression Anxiety Stress Scales (DASS-Anxiety), a 14-item instrument assessing autonomic arousal, situational anxiety, and subjective anxiety symptoms [40]. Participants rate the extent to which each statement applied to them over the past week on a 4-point scale ranging from 0 (“did not apply to me at all”) to 3 (“applied to me very much or most of the time”). Higher scores reflect greater anxiety symptom severity. The DASS-Anxiety subscale has demonstrated strong internal consistency and convergent validity [40]. Internal consistency in the current sample at each time point was as follows: α = 0.90 (pretreatment), 10-week follow-up (α = 0.91), and 14-week follow-up (α = 0.90).

Physical health symptoms were measured using the Patient Health Questionnaire-15 (PHQ-15), a 15-item measure evaluating common physical symptoms [41]. Participants rate how much they were bothered by symptoms (e.g., stomach pain, fatigue, dizziness) during the past four weeks on a 3-point scale from 0 (“not bothered at all”) to 2 (“bothered a lot”). Higher scores indicate greater somatic symptom burden. The PHQ-15 has demonstrated good reliability and criterion validity [41]. Internal consistency in the current sample at each time point was as follows: α = 0.80 (pretreatment), 10-week follow-up (α = 0.73), and 14-week follow-up (α = 0.81).

Resilience was assessed using the 10-item Connor–Davidson Resilience Scale (CD-RISC-10), which measures the ability to cope with stress and adversity [42]. Items (e.g., “I am able to adapt when changes occur”) are rated on a 5-point scale ranging from 0 (“not true at all”) to 4 (“true nearly all the time”). Items are summed with higher scores indicate greater resilience. The CD-RISC-10 has demonstrated strong reliability and validity [42,43]. Internal consistency in the current sample at each time point was as follows: α = 0.84 (pretreatment), 10-week follow-up (α = 0.88), and 14-week follow-up (α = 0.90).

### 2.5. Intervention: RISE

RISE is a flexible, survivor-centered psychosocial intervention that consisted of up to six sessions delivered over 10 weeks for the purposes of the RCT (see Iverson et al., 2021 [29], for more details). To promote choice, voice, and empowerment, participants selected from six modules based on their priorities for each session. Modules can be completed in any order and can be repeated as desired, and included: safety planning; IPV health effects and warning signs; coping and self-care; enhancing social support; making difficult decisions; and connecting with resources and moving forward. Sessions incorporated handouts, structured exercises, and motivational interviewing techniques to enhance readiness for change and goal pursuit. Participants are invited to set behaviorally specific SMART goals (specific, measurable, achievable, realistic, and time-limited) that are aligned with their personal values that relate to the modules, and discuss and problem-solve potential barriers to accomplishing their goals. Participants determined whether to schedule additional sessions (up to the six-session limit). Participants were referred to community and VHA services as was relevant and desired by the patient.

### 2.6. Intervention: ECAU

ECAU was a single 60 min advocacy-based session that included best practices for care as usual in VHA (see Iverson et al., 2021 [29]). This intervention includes psychoeducation regarding IPV (i.e., types, prevalence, health impacts), validation and supportive responses, safety planning, local and national resources, and service referrals in and outside VHA.

### 2.7. Statistical Analysis

We used IBM SPSS Version 29 for these analyses. To account for any baseline differences between women with and without a history of probable IPV-related TBI, independent t-tests and chi-square analyses were first conducted. If baseline differences were found, pretreatment scores for that outcome were included as covariates within multilevel modeling analyses, with the outcome consisting only of the two later time points to avoid statistical redundancy and violation of model assumptions (i.e., independent residuals). Subsequently, multilevel modeling analyses using the SPSS mixed procedure were conducted to examine changes in self-reported outcomes within each treatment condition (i.e., RISE, ECAU), within and across IPV-related TBI groups (i.e., with versus without probable IPV-related TBI), and the interaction of both treatment condition and probable IPV-related TBI status. This statistical approach is appropriate for secondary analyses of clinical trial data, including trials with small samples, and provides corresponding statistical corrections [44,45,46], while using all available data (to account for missing data) irrespective of whether participants completed all timepoints for assessment [47].

Unconditional change models were conducted to identify if linear or nonlinear change models were more appropriate for the data structure. Multilevel modeling is an iterative process, and as such, smaller −2 log likelihood (i.e., deviance) values combined with other fit statistics (i.e., Akaike Information Criterion [AIC], Bayesian Information Criterion [BIC]) were used to determine the best fitting model for the data. *p*-values of less than 0.05 were used to determine statistical significance. Effect sizes (d) for changes within each group of women and change between the two groups were calculated using Feingold’s procedures [48] for effect size estimates from growth curve analyses, which are comparable to those from traditional repeated measures designs. Treatment condition, time, probable IPV-related TBI status, and their interaction were fixed effects, and the intercept was a random effect to account for repeated observations within participants. Restricted maximum likelihood (REML) was used as the estimation method for all models, and degrees-of-freedom were estimated using the Satterthwaite approximation.

## 3. Results

### 3.1. Preliminary Analyses

As reported by Pebole et al. (2024) [30], approximately one third of the total sample (35%) had a history of probable IPV-related TBI (N = 21; ECAU = 10, RISE = 11). The one participant dropped from analyses did not have a history of probable IPV-related TBI at pretreatment. See Iverson et al.’s work (2021) [29] for the CONSORT flow diagram for this clinical trial. There were no significant differences between women with and without a history of probable IPV-related TBI at pretreatment in terms of sociodemographic characteristics (e.g., age, race, ethnicity; see Table 1 for descriptive statistics). There were no significant group differences at pretreatment for self-efficacy, empowerment, patient activation, valued living, depressive symptoms, or resilience (see Table 1). However, women with a history of probable IPV-related TBI had significantly higher physical health symptoms, *t*(49) = −3.15, *p* = 0.003, and anxiety symptoms, *t*(56) = −2.65, *p* = 0.01, in comparison to women without probable IPV-related TBI histories.

### 3.2. Effect of Time

Unconditional multilevel models suggested that modeling of variables beyond time and/or non-linear time on all outcomes of interest was warranted. Linear change was determined for each of these outcomes prior to primary analyses. See Table 2 for mean scores for each of our outcomes’ measures at each assessment time point for both groups.

### 3.3. Primary Analyses

See Table 3 for detailed results of analyses with probable IPV-related TBI history status as a predictor of change in outcomes. Time was categorically coded (i.e., pretreatment, 10-week follow-up, 14-week follow-up; intercept was pre-treatment) and a linear function was included in the models. The covariance structure for each outcome was as follows: self-efficacy (random effects = variance components; time = autoregressive moving average), empowerment (random effects = variance components; time = first-order autoregressive), patient activation (random effects = variance components; time = unstructured), valued living (random effects = variance components; time = first-order ante-dependence), physical health symptoms (random effects = variance components; time = heterogeneous Toeplitz), depression (random effects = variance components; time = Huynh–Feldt), anxiety (random effects = variance components; time = diagonal), and resilience (random effects = variance components; time = first-order autoregressive).

**Figure 1:**
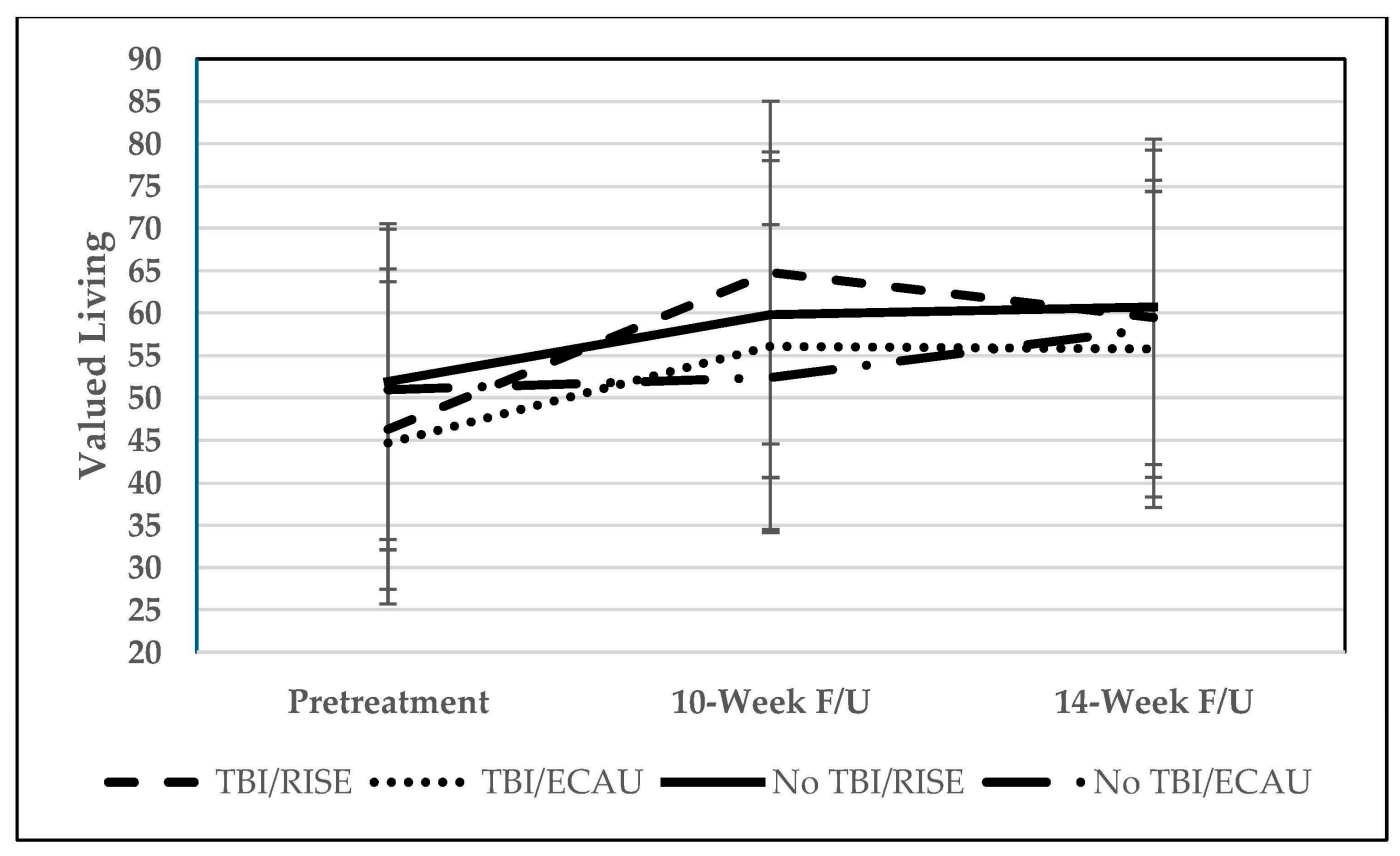
Estimated marginal means for primary outcomes by treatment condition and probable IPV-related TBI status at each time point for valued living. Note: F/U = Follow-up; TBI = IPV-related Traumatic Brain Injury; RISE = Recovering from Intimate Partner Violence through Strengths and Empowerment; and ECAU = Enhanced Care As Usual.

**Figure 2:**
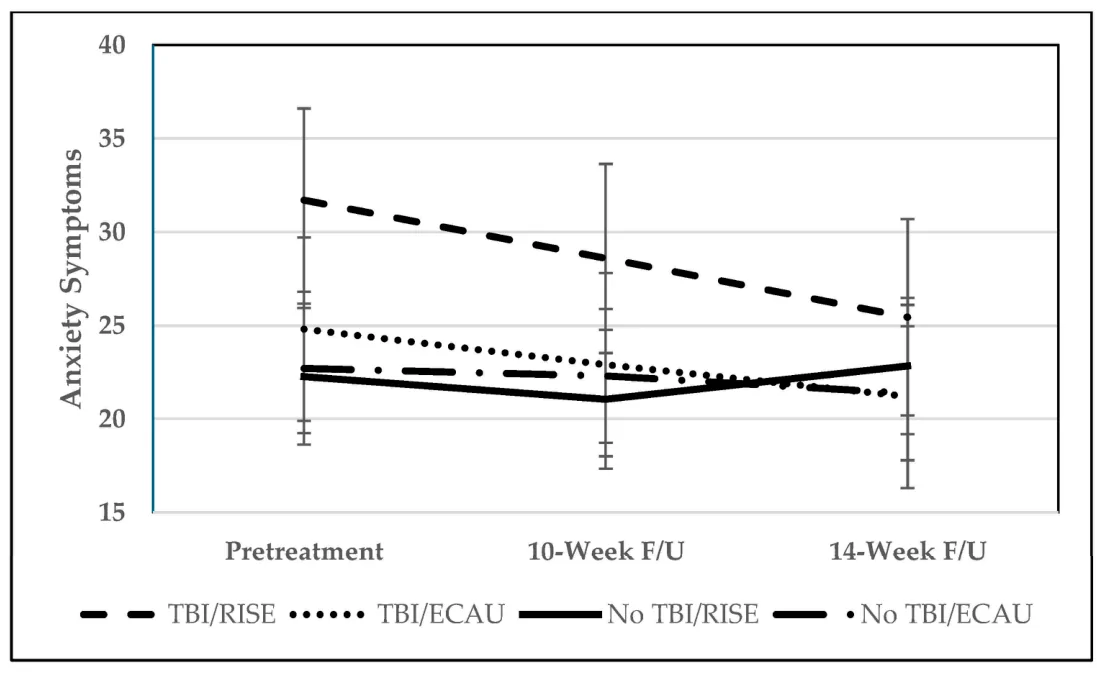
Estimated marginal means for primary outcomes by treatment condition and probable IPV-related TBI status at each time point for anxiety symptoms. Note: F/U = Follow-up; TBI = IPV-related Traumatic Brain Injury; RISE = Recovering from Intimate Partner Violence through Strengths and Empowerment; and ECAU = Enhanced Care As Usual.

There were no significant probable IPV-related TBI main effects or interaction effects for several of our outcomes, including self-efficacy, empowerment, patient activation, depressive symptoms, or resilience (*p* > 0.05). Nonetheless, there was a significant interaction effect of probable IPV-related TBI status and pretreatment physical health symptoms for: physical health symptoms as an outcome, *F*(1, 134) = 8.99, *p* = 0.004; interaction effects of time and probable IPV-related TBI history for valued living *F*(2, 76.55) = 2.96, *p* = 0.049 (see Figure 1); and interaction effects of probable IPV-related TBI history status, treatment condition, and pretreatment anxiety symptoms for anxiety symptoms as an outcome, *F*(1, 48) = 5.04, *p* = 0.029 (see Figure 2). See Supplementary Table S1 for detailed information on estimates of fixed effects across outcomes. 

## 4. Discussion

This study examined whether a history of probable IPV-related TBI moderated treatment response on psychosocial outcomes across two brief healthcare-based counseling interventions for women experiencing past-year IPV. Overall, findings indicate that probable IPV-related TBI history was not a significant moderator of treatment response across most psychosocial outcomes examined. Specifically, no significant interaction effects involving probable IPV-related TBI history emerged for five of the eight psychosocial domains we examined in this study, including self-efficacy, empowerment, patient activation, depression, and resilience. However, three notable exceptions were observed. In particular, results demonstrated different pathways regarding valued living; women with histories of probable IPV-related TBI demonstrated more dramatic improvement in valued living (from pretreatment to the first follow-up) in comparison to a smaller increase (15 points vs. 5 points on average) for women without these histories. Additionally, women with probable IPV-related TBI history in the RISE condition demonstrated higher anxiety symptoms on average at each time point (i.e., cross-sectional differences) in comparison to women without a history of probable IPV-related TBI in the RISE condition (no differences were found between groups for the ECAU condition); this is not entirely unexpected as women with probable IPV-related TBI histories had significantly more severe anxiety symptoms at pretreatment, which was also a significant covariate. Lastly, women with probable IPV-related TBI histories had significantly more physical health symptoms at pretreatment (also a significant covariate), with probable IPV-related TBI history predicting physical health outcomes at each of the three time points cross-sectionally, but not its change over time. This finding was not surprising as women with probable IPV-related TBI histories likely report worse physical health globally as a downstream effect of these experiences, made evident by the consistently higher scores at each time point. These findings nonetheless suggest that probable IPV-related TBI history did not broadly moderate treatment response across most psychosocial outcomes examined. At the same time, the observed differences in valued living, anxiety, and physical health highlight specific domains that warrant further investigation in future studies.

Across most psychosocial domains examined, the results did not detect statistically significant differences in treatment response between women with and without probable IPV-related TBI history. Accordingly, probable IPV-related TBI history was not a significant moderator of response to either advocacy-based ECAU or RISE across these core domains of functioning, although the modest sample size likely limited our ability to detect more subtle differences. Despite concerns in the prior literature that cognitive and neurological sequalae associated with TBI could potentially interfere with response to psychosocial treatment [20,27,28,49], the present findings did not detect significant differences in improvements between women with and without probable IPV-related TBI history across multiple key domains of recovery (i.e., self-efficacy, empowerment, patient activation, depression, or resilience). This pattern aligns with emerging work suggesting that, when interventions are accessible and strengths-based, individuals with a history of probable IPV-related TBI may be able to meaningfully engage in treatment, although additional research with larger samples is needed to confirm these findings [30,50,51]. Collectively, these findings suggest that probable IPV-related TBI history did not emerge as a significant moderator of psychosocial treatment response across most outcomes examined.

The observed interaction between time and probable IPV-related TBI history for valued living suggests a more nuanced pattern of treatment response on this outcome regardless of treatment condition. Specifically, women with probable IPV-related TBI histories appear to exhibit a steeper rise in this outcome. Although this finding is intriguing, it should be interpreted cautiously given the exploratory nature of the analyses and the relatively small subgroup of women with probable IPV-related TBI. One possible, but untested, explanation is that valued living, which refers to engaging in behaviors that are aligned with one’s personal values, may rely on executive functioning processes, including planning, self-regulation, cognitive flexibility and sustained goal-directed behavior, all of which are necessary for translating abstract values into consistent action over time [52,53]. Because these cognitive processes were not directly measured in the present study, this interpretation remains speculative. Although treatment response did not significantly differ according to probable IPV-related TBI history in domains such as empowerment and self-efficacy, valued living may represent a more behaviorally anchored construct that reflects the extent to which individuals are able to enact these value-consistent behaviors routinely in daily life over time [38]. Accordingly, one possible explanation is that women with a history of probable IPV-related TBI may experience greater improvements in valued living during treatment because intervention support may help compensate for cognitive challenges that could otherwise interfere with consistently enacting value-congruent behaviors. However, this interpretation remains speculative because these cognitive processes were not assessed. Additionally, the interaction with time suggests that the observed pattern may reflect the transition from structured intervention support to independently initiating, organizing, and sustaining value-consistent behaviors in everyday life. Since these activities may rely on executive functioning processes, they may become more challenging following treatment completion for some individuals with probable IPV-related TBI history [54], although this hypothesis was not directly tested. This was reflected by the data in the women with probable IPV-related TBI histories showing a subsequent slight decrease from the 10-week to 14-week follow-up (i.e., after completion of treatment), whereas women without probable IPV-related TBI histories continued to improve in valued living. Overall, these findings may suggest additional structure may be needed to support sustained valued action among individuals with probable IPV-related TBI history (e.g., skills to enhance executive functioning [planning, initiation, organization, and follow-through]; preventing cognitive fatigue; and/or setting reminders).

A significant interaction between treatment condition and probable IPV-related TBI history was observed for anxiety symptoms. Specifically, women with probable IPV-related TBI in the RISE condition reported higher anxiety symptoms on average across the study period than women without probable IPV-related TBI in the RISE condition (and ECAU participants with and without probable IPV-related TBI histories) at each individual time point, even with declines in pretreatment symptoms among those with probable IPV-related TBI histories in the RISE group at both follow-up assessments. Because a significant interaction with time was not observed, these findings should be interpreted as reflecting differences in symptom levels across groups rather than differential change over time. One possible explanation is that the more reflective and empowerment-focused nature of RISE may increase participants’ awareness of ongoing stressors, safety concerns, and functional challenges. However, because participants’ awareness of these experiences was not directly assessed, this interpretation is uncertain. For some individuals with a history of probable IPV-related TBI, this heightened awareness of emotional, cognitive, and functional difficulties may contribute to higher levels of anxiety, even in the context of the broader therapeutic benefit on other important psychosocial outcomes [55]. Relatedly, it is possible that RISE may place greater cognitive and emotional demands on participants, including behaviorally specific goal setting, self-reflection, and integration of new skills (e.g., coping strategies, making difficult decisions). Individuals with a history of TBI who experience difficulties in attention, executive functioning, or emotional regulation may find these processes more effortful, potentially contributing to increased anxiety over time [56,57]. Although this interpretation is consistent with the prior literature, the proposed mechanisms were not directly evaluated in the present study and therefore warrant empirical investigation in future research. Another possible explanation is that the consistently elevated anxiety observed among participants with probable IPV-related TBI history in the RISE condition may reflect engagement in meaningful but emotionally demanding behavioral changes, such as setting interpersonal boundaries, accessing resources, enacting specific safety plans, or reevaluation of intimate and social relationships. These shifts are likely adaptive but may be anxiety-provoking, particularly in the context of ongoing IPV risk, ongoing stress, and TBI-related cognitive, emotional, and behavioral vulnerabilities. In contrast, advocacy-based approaches, such as the ECAU intervention examined in this study, may involve more concrete, externally supported, or immediately pragmatic assistance that may be easier to sustain despite cognitive impairments, resulting in no differences between women with and without probable IPV-related TBI histories within this treatment condition. Advocacy-based approaches may rely less heavily on independent execution of reflective or skills-based strategies, and more on linkages, validation, and tangible support. Finally, it is important to note that outcomes were assessed only 10 and 14 weeks after enrollment (i.e., approximately posttreatment and only one-month following treatment completion for the RISE participants), which may represent a relatively brief window to capture longer-term adjustment in anxiety symptoms (hence the nonsignificant effect of time). Although the present study did not detect differential change in anxiety over time, it is possible that these higher anxiety levels reflect a transient response that may attenuate over longer follow-up periods as participants consolidate and integrate RISE-related skills and experience increased stability. Indeed, research suggests that improvements in empowerment and self-efficacy, which were evident among this group of women with probable IPV-related TBI history in the RISE condition, can lead to improvements in mental health and well-being over time [58,59].

These findings have preliminary implications for the future development and refinement of healthcare-based IPV interventions. A key takeaway is that we did not observe evidence that probable IPV-related TBI broadly limited response to these brief, trauma-informed IPV interventions. At the same time, the identified differences in specific domains suggest the possibility that brain-injury informed adaptations may enhance long-term treatment responsiveness in terms of targeting valued living and anxiety symptoms for some individuals with a probable IPV-related TBI history. Although not evaluated in the present study, clinicians delivering IPV interventions to women with probable IPV-related TBI history may benefit from incorporating cognitive accommodations such as simplified materials, incorporating repetition of key concepts, and visual cues to support more pervasive comprehension and retention [60,61]. Clinicians may also benefit from remaining mindful about potential difficulties in emotion regulation, attention, memory, executive functioning, and language abilities, including verbal fluency and receptive language, which may influence treatment engagement and skill application [62,63]. These considerations align closely with principles of trauma-informed care, which emphasize safety, flexibility, and responsiveness to individual needs, and highlight the added benefits of incorporating a brain injury-informed lens into intervention delivery [64,65].

This study should be interpreted in light of several limitations and considerations. Participants were women veterans receiving care within the VHA. Consequently, the findings may not generalize to civilian women who have experienced IPV, individuals receiving care in non-VHA or less integrated healthcare settings, or other populations with TBI. The sample size was modest, which may have limited statistical power to detect interaction effects, particularly more nuanced differences in treatment response by history of probable IPV-related TBI status. The study was not powered to detect treatment moderation effects, so nonsignificant interaction findings should not be taken as evidence of treatment equivalence. In addition, our subsample of women who experienced probable IPV-related TBI history was too small to examine additional important questions about whether there are differences in treatment responsiveness based on persistent and current symptoms from their TBI(s) (i.e., cognitive and somatosensory symptoms), which could impact IPV intervention helpfulness. Furthermore, because the study involved multiple comparisons across several psychosocial outcomes, the probability of Type I error is increased, and the few statistically significant findings should be interpreted as exploratory. Additionally, probable IPV-related TBI history was assessed via self-report screening rather than a comprehensive diagnostic evaluation (e.g., structured clinical interview or a validated lifetime TBI assessment), introducing the potential for misclassification and recall bias. Furthermore, although the modified VA TBI screening instrument has been used in previous studies of women veterans and aligns with VA-recommended screening tools, it has not been formally validated, which may have influenced participant classification and, consequently, the study findings. Relatedly, important injury characteristics were not captured, including severity, recency, number of TBIs and persistent concussive symptoms, all of which may meaningfully influence cognitive, emotional, and treatment-related outcomes [66,67]. Additionally, this study focused on probable IPV-related TBI history and did not assess repetitive head impacts or cumulative head impact exposure, limiting our ability to evaluate how repetitive head trauma may contribute to psychosocial outcomes and intervention response. The absence of these contextual factors limits the ability to fully characterize the role of probable IPV-related TBI history in shaping intervention response. Finally, the follow-up period was brief, precluding examination of longer-term trajectories of psychosocial functioning and durability of treatment responsiveness among women with a history of probable IPV-related TBI.

Future research should address these limitations by conducting larger, adequately powered samples. Furthermore, comprehensive and validated TBI assessment methods, such as the BATL-IPV interview [23] or the Brain Injury Screening Questionnaire IPV Module [68], could be helpful for accurately characterizing injury history and capturing key features such as severity, frequency, recency, cumulative exposure to repetitive head impacts, mechanisms of injury (e.g., blunt force and non-fatal strangulation), and acute injury characteristics (e.g., loss or alteration of consciousness and post traumatic amnesia). In addition, examining potential mechanisms, including cognitive functioning, executive control, and emotional regulation, may help clarify pathways through which TBI history influences treatment response and identify targets for further intervention. This work may also inform the development of more tailored approaches that better accommodate TBI-related challenges within IPV interventions. Although the interventions evaluated in the present study were not specifically adapted to address cognitive sequelae associated with probable IPV-related TBI, future research should examine whether incorporating brain injury-informed accommodations, such as modifying the pace of intervention delivery, reinforcing key concepts, allowing additional processing time and breaks for cognitive fatigue, and incorporating compensatory strategies, improves psychosocial outcomes [69]. Additionally, research is needed to help clinicians better identify which women with IPV-related TBI histories would benefit from more brain injury-informed interventions following implementation of IPV-specific interventions. Moreover, extending this research beyond the VHA settings to include civilian women who have experienced IPV, as well as individuals receiving care in community-based and other healthcare settings, will be important for determining the generalizability of these findings across diverse populations and healthcare systems. Extended follow-up intervals will be critical for clarifying the impact of probable IPV-related TBI history on intervention outcomes. Future research is needed to determine whether similar patterns of findings are observed among individuals who sustain TBI through other mechanisms and within more diverse populations. Additionally, future research should examine whether these findings generalize across culturally diverse populations, as cultural differences in the expression and reporting of psychosocial symptoms may influence the validity and interpretation of self-report measures.

## 5. Conclusions

In sum, the present findings suggest that probable IPV-related TBI history was not a moderator of most psychosocial outcomes from brief, healthcare-based IPV interventions. At the same time, observed differences in valued living and anxiety symptoms highlight potentially important areas where TBI-related cognitive, emotional, and behavioral processes may shape treatment response. As recognition of probable IPV-related TBI continues to grow, these findings underscore the importance of further investigating how brain injury-informed considerations can be integrated into trauma-informed care to optimize outcomes.

## Figures and Tables

**Table 1:** Descriptive statistics by probable IPV-related TBI history status.

Sociodemographic Characteristic	Probable IPV-Related TBI Hx (n = 21)	No Probable IPV-Related TBI Hx (n = 39)	Statistic	*p* Value
**Age, mean (SD)**	36.4 (8.9)	40.5 (13.2)	*t* = 1.42	0.16
**Race**			c^2^ = 2.62	0.45
Black	4 (19.0)	9 (23.1)		
White	12 (57.1)	21 (53.8)		
Asian	1 (4.8)	1 (2.6)		
Mixed Race	2 (9.5)	5 (12.8)		
Other Race	2 (9.5)	3 (7.7)		
**Ethnicity (Hispanic)**	4 (19.0)	8 (20.5)	c^2^ = 0.02	0.89
**Sexual Orientation**			c^2^ = 1.04	0.79
Heterosexual	14 (66.7)	29 (74.4)		
Lesbian/Gay	1 (4.8)	3 (7.7)		
Bisexual	4 (19.0)	5 (12.8)		
Pansexual	2 (9.5)	2 (5.1)		
**Marital Status**			c^2^ = 4.55	0.47
Married/Living Together	3 (14.3)	9 (23.1)		
Married/ Not Living Together	6 (28.6)	5 (12.8)		
Not Married/ Not Living Together	1 (4.8)	3 (7.7)		
Living Together/ Not Married	3 (14.3)	9 (23.1)		
Single	5 (23.8)	11 (28.2)		
Separated	3 (14.3)	2 (5.1)		
Military Branch			c^2^ = 7.71	0.17
Army	15 (71.4)	16 (41.0)		
Navy	0 (0)	7 (17.9)		
Air Force	3 (14.3)	5 (12.8)		
Marine Corps	3 (14.3)	6 (15.4)		
Reservists	0 (0)	1 (2.6)		
National Guard	0 (0)	2 (5.1)		
**Pretreatment Outcomes, mean (SD)**				
Self-efficacy (GSES)	29.14 (5.62)	29.38 (4.73)	*t* = 0.18	0.86
Empowerment (PPS-R)	127.38 (27.51)	133 (20.20)	*t* = 0.90	0.37
Patient Activation (PAM-13)	59.79 (13.71)	68.20 (18.90)	*t* = 1.98	0.053
Valued Living (VLQ)	45.21 (17.74)	51.63 (17.88)	*t* = 1.33	0.19
**Physical Health Sxs (PHQ-15)**	**14.17 (4.78)**	**9.58 (5.07)**	***t* = −3.15**	**0.003**
Depressive Sxs (CES-D)	28.80 (12.73)	23.83 (10.58)	*t* = −1.54	0.13
**Anxiety Sxs** **(DASS-Anxiety)**	**28.25 (9.76)**	**22.50 (6.65)**	***t* = −2.65**	**0.01**
Resilience (CD-RISC-10)	24.80 (6.53)	24.74 (5.87)	*t* = −0.03	0.97

Note: IPV = Intimate Partner Violence; TBI = Traumatic Brain Injury; Hx = History; GSES = General Self-Efficacy Scale; PPS-R = Personal Progress Scale-Revised; PAM-13 = Patient Activation Measure; VLQ = Valued Living Questionnaire; PHQ-15 = Patient Health Questionnaire-15; CES-D = Center for Epidemiologic Studies Depression Scale; DASS-Anxiety = Depression Anxiety Stress Scales—Anxiety Subscale; CD-RISC-10 = Connor–Davidson Resilience Scale; Sxs = symptoms. Bolded text indicates statistical significance.

**Table 2:** Outcome measures at pretreatment and follow-up assessments.

Scale	Pretreatment	10-Week Follow-Up	14-Week Follow-Up
**PPS-R**			
Probable IPV-related TBI Hx			
RISE	129.08 (115.69 to 142.47)	144.91 (132.21 to 157.61)	141.83 (129.45 to 154.21)
ECAU	129.70 (113.67 to 145.73)	136.80 (122.95 to 150.65)	139.60 (126.78 to 152.42)
No Probable IPV-related TBI Hx			
RISE	130.25 (118.42 to 140.92)	144.42 (134.44 to 153.74)	149.83 (140.59 to 158.89)
ECAU	133.52 (122.18 to 145.35)	138.45 (128.58 to 148.32)	137.66 (128.44 to 146.88)
**GSES**			
Probable IPV-related TBI Hx			
RISE	29.42 (19.17 to 39.67)	32.70 (23.47 to 41.93)	33.94 (22.42 to 45.46)
ECAU	29.30 (20.16 to 38.44)	29.90 (22.56 to 37.24)	30.50 (18.28 to 42.72)
No Probable IPV-related TBI Hx			
RISE	28.28 (20.36 to 36.20)	33.03 (24.47 to 41.59)	34.04 (23.69 to 44.41)
ECAU	30.20 (21.13 to 39.27)	31.12 (21.79 to 40.45)	30.82 (21.88 to 39.76)
**PAM-13**			
Probable IPV-related TBI Hx			
RISE	62.10 (46.35 to 77.86)	72.64 (52.46 to 92.81)	70.75 (48.31 to 93.18)
ECAU	58.40 (37.60 to 79.20)	65.37 (42.86 to 87.88)	65.98 (49.54 to 82.42)
No Probable IPV-related TBI Hx			
RISE	68.08 (42.87 to 93.29)	77.72 (65.92 to 89.52)	78.27 (60.34 to 96.20)
ECAU	67.74 (49.87 to 85.60)	71.62 (52.08 to 91.17)	71.60 (60.85 to 82.35)
**VLQ**			
Probable IPV-related TBI Hx			
RISE	46.31 (27.41 to 65.21)	64.79 (44.57 to 85.01)	59.44 (38.32 to 80.56)
ECAU	44.70 (25.69 to 63.71)	56.08 (34.13 to 78.03)	55.72 (37.11 to 74.33)
No Probable IPV-related TBI Hx			
RISE	51.94 (33.34 to 70.54)	59.83 (40.62 to 79.04)	60.72 (42.17 to 79.27)
ECAU	50.97 (32.06 to 69.88)	52.46 (34.51 to 70.41)	58.17 (40.63 to 75.71)
**PHQ-15**			
Probable IPV-related TBI Hx			
RISE	15.23 (10.91 to 19.55)	14.47 (9.23 to 19.71)	12.13 (8.87 to 15.39)
ECAU	11.39 (6.73 to 16.05)	13.81 (9.97 to 17.65)	9.44 (6.29 to 12.61)
No Probable IPV-related TBI Hx			
RISE	11.38 (7.83 to 14.93)	9.92 (6.39 to 13.45)	9.57 (5.12 to 14.02)
ECAU	11.36 (7.98 to 14.74)	11.49 (6.79 to 16.19)	10.00 (5.56 to 14.44)
**CES-D**			
Probable IPV-related TBI Hx			
RISE	29.14 (19.17 to 39.11)	24.46 (17.29 to 31.63)	26.99 (19.77 to 34.21)
ECAU	27.10 (18.65 to 35.55)	22.40 (14.17 to 30.63)	22.20 (16.89 to 27.51)
No Probable IPV-related TBI Hx			
RISE	26.08 (17.81 to 34.35)	17.91 (13.44 to 22.38)	18.33 (15.51 to 21.15)
ECAU	23.20 (16.21 to 30.19)	22.56 (18.11 to 27.01)	18.19 (13.16 to 23.22)
**DASS-Anxiety**			
Probable IPV-related TBI Hx			
RISE	31.70 (26.8 to 36.6)	28.58 (23.52 to 33.65)	25.44 (20.19 to 30.70)
ECAU	24.80 (19.9 to 29.7)	22.90 (18 to 27.8)	21.20 (16.3 to 26.1)
No Probable IPV-related TBI Hx			
RISE	22.28 (18.63 to 25.93)	21.05 (17.34 to 24.77)	22.83 (19.18 to 26.49)
ECAU	22.70 (19.24 to 26.17)	22.30 (18.72 to 25.88)	21.37 (17.79 to 24.95)
**CD-RISC-10**			
Probable IPV-related TBI Hx			
RISE	25.30 (15.14 to 35.46)	26.37 (16.18 to 36.56)	28.40 (18.14 to 38.65)
ECAU	24.30 (14.14 to 34.46)	26.10 (16.22 to 35.78)	25.97 (16.19 to 35.76)
No Probable IPV-related TBI Hx			
RISE	25.53 (15.73 to 35.32)	30.83 (21.02 to 40.65)	29.99 (20.16 to 39.81)
ECAU	24 (14.23 to 33.78)	25.98 (16.22 to 35.78)	25.97 (23.09 to 28.86)

Note. IPV = Intimate Partner Violence; TBI = Traumatic Brain Injury; Hx = History; RISE = Recovering from IPV through Strengths and Empowerment; ECAU = Enhanced Care as Usual; PPS-R = Personal Progress Scale-Revised; GSES = General Self-Efficacy Scale; PAM-13 = Patient Activation Measure; VLQ = Valued Living Questionnaire; PHQ-15 = Patient Health Questionnaire-15; CES-D = Center for Epidemiologic Studies Depression Scale; DASS-Anxiety = Depression Anxiety Stress Scales—Anxiety Subscale; CD-RISC-10 = Connor–Davidson Resilience Scale.

**Table 3:** Multilevel modeling fixed effects results.

Model	F	*p*-Value	Between Group Effect Size
**PPS-R (Empowerment)**			−0.13
Probable IPV-related TBI Status	0.15	0.7	
Tx Condition	0.56	0.46	
**Time**	**12.58**	**<0.001**	
Condition * Probable IPV-related TBI Hx	0.03	0.87	
Time * Probable IPV-related TBI Hx	0.22	0.8	
Condition * Time	2.23	0.11	
Condition * Time * Probable IPV-related TBI Hx	0.97	0.38	
**GSES (Self-Efficacy)**			−0.04
Probable IPV-related TBI Hx	0.07	0.79	
Tx Condition	2.06	0.16	
**Time**	**13.29**	**<0.001**	
Condition * Probable IPV-related TBI Hx	0.24	0.63	
Time * Probable IPV-related TBI Hx	0.42	0.66	
Condition * Time	**6.34**	**0.003**	
Condition * Time * Probable IPV-related TBI Hx	0.26	0.78	
**PAM-13 (Patient Activation)**			−0.37
Probable IPV-related TBI Hx	2.85	0.1	
Tx Condition	1.78	0.19	
**Time**	**6.49**	**0.003**	
Condition * Probable IPV-related TBI Hx	0.01	0.91	
Time * Probable IPV-related TBI Hx	0.1	0.9	
Condition * Time	0.53	0.6	
Condition * Time * Probable IPV-related TBI Hx	0.21	0.81	
**VLQ (Valued Living)**			**−0.10**
Probable IPV-related TBI Hx	0.07	0.79	
Tx Condition	0.89	0.35	
**Time**	**8.11**	**<0.001**	
Condition * Probable IPV-related TBI Hx	0.02	0.9	
Time * Probable IPV-related TBI Hx	**2.96**	**0.049**	
Condition * Time	1.38	0.26	
Condition * Time * Probable IPV-related TBI Hx	0.002	0.998	
**PHQ-15 (Physical Health Symptoms)**			0.58
**Probable IPV-related TBI Hx**	**4.52**	**0.013**	
Probable IPV-related TBI Hx * Pretreatment PHS	**8.99**	**0.004**	
Tx Condition	0.76	0.38	
**Time**	**4.83**	**0.011**	
Time * Pretreatment PHS	**5.67**	**0.004**	
**Pretreatment PHS**	**151.15**	**<0.001**	
Condition * Probable IPV-related TBI Hx	0.05	0.83	
Time * Probable IPV-related TBI Hx	2.01	0.14	
Condition * Time	0.86	0.42	
Condition * Time * Probable IPV-related TBI Hx	0.16	0.86	
**CES-D (Depressive Symptoms)**			0.56
Probable IPV-related TBI Hx	0.01	0.92	
Tx Condition	2.07	0.15	
Time	2.13	0.12	
Condition * Probable IPV-related TBI Hx	0.57	0.45	
Time * Probable IPV-related TBI Hx	0.18	0.84	
Condition * Time	1.16	0.32	
Condition * Time * Probable IPV-related TBI Hx	0.42	0.66	
**DASS-Anxiety (Anxiety Symptoms)**			**0.20**
**Probable IPV-related TBI Hx**	**4.03**	**0.037**	
**Tx Condition**	**3.17**	**0.049**	
Time	2.17	0.15	
**Pretreatment Anxiety**	**58.54**	**<0.001**	
Condition * Probable IPV-related TBI Hx	1.15	0.288	
Condition * Probable IPV-related TBI Hx *Anxiety	**5.041**	**0.029**	
Time * Probable IPV-related TBI Hx	1.63	0.19	
**CD-RISC-10 (Resilience)**			−0.09
Probable IPV-related TBI Hx	0.35	0.56	
Tx Condition	2.2	0.14	
**Time**	**8.21**	**<0.001**	
Condition * Probable IPV-related TBI Hx	0.6	0.44	
Time * Probable IPV-related TBI Hx	1.84	0.17	
Condition * Time	0.61	0.55	
Condition * Time * Probable IPV-related TBI Hx	1.42	0.25	

Note: * indicates interaction terms; IPV-related TBI Hx = Intimate Partner Violence-related TBI history; PPS-R = Personal Progress Scale-Revised; GSES = General Self-Efficacy Scale; PAM-13 = Patient Activation Measure; VLQ = Valued Living Questionnaire; PHQ-15 = Patient Health Questionnaire-15; CES-D = Center for Epidemiologic Studies Depression Scale; DASS-Anxiety = Depression Anxiety Stress Scales—Anxiety Subscale; and CD-RISC-10 = Connor–Davidson Resilience Scale. Bolded text indicates statistical significance.

## Data Availability

The data presented in this study are available on request from the corresponding author due to privacy restrictions.

## Supplementary Materials

The following supporting information can be downloaded at: https://www.mdpi.com/article/10.3390/brainsci16080836/s1, Table S1: Detailed information on estimates of fixed effects across outcomes.
